# Improved prediction of missing protein interactome links via anomaly detection

**DOI:** 10.1007/s41109-017-0022-7

**Published:** 2017-01-28

**Authors:** Kushal Veer Singh, Lovekesh Vig

**Affiliations:** School of Computational and Integrative Sciences, Jawaharlal Nehru University, New Delhi, Delhi, India

**Keywords:** Link prediction, Anomaly detection, Protein protein interaction networks

## Abstract

Interactomes such as Protein interaction networks have many undiscovered links between entities. Experimental verification of every link in these networks is prohibitively expensive, and therefore computational methods to direct the search for possible links are of great value. The problem of finding undiscovered links in a network is also referred to as the link prediction problem. A popular approach for link prediction has been to formulate it as a binary classification problem in which class labels indicate the existence or absence of a link (we refer to these as positive links or negative links respectively) between a pair of nodes in the network. Researchers have successfully applied such supervised classification techniques to determine the presence of links in protein interaction networks. However, it is quite common for protein-protein interaction (PPI) networks to have a large proportion of undiscovered links. Thus, a link prediction approach could incorrectly treat undiscovered positive links as negative links, thereby introducing a bias in the learning. In this paper, we propose to denoise the class of negative links in the training data via a Gaussian process anomaly detector. We show that this significantly reduces the noise due to mislabelled negative links and improves the resulting link prediction accuracy. We evaluate the approach by introducing synthetic noise into the PPI networks and measuring how accurately we can reconstruct the original PPI networks using classifiers trained on both noisy and denoised data. Experiments were performed with five different PPI network datasets and the results indicate a significant reduction in bias due to label noise, and more importantly, a significant improvement in the accuracy of detecting missing links via classification.

## Introduction

Graphical networks can depict many complex systems involving biological, social and informational connections between entities. At the most abstract level, these networks are modelled by graphs in which nodes represent individuals or agents and links denote the interactions or relationships between nodes. Structural properties of biological networks are of great interest as they directly correlate with biological function (Qi and Ge 2006; Wuchty et al. 2003). Various attempts have been made to understand the topological evolution of networks (Albert and Barabási 2002; Dorogovtsev and Mendes 2002). The evolution of networks involves two processes: i) the addition or deletion of nodes and ii) The addition or deletion of edges (links) between nodes. The second process of topological evolution particularly when new connections are added to the existing network has not yet been concretely formalised and revolves around the link-prediction problem. Many applications utilize link prediction to identify new links in large, sparse networks armed only with knowledge of network topology. Therefore, improvements in link prediction accuracy will be of great significance in both science and engineering applications. Meanwhile, link-prediction also reflects the extent to which the evolution of a network can be modelled by topological features intrinsic to the network itself.

The link prediction task can be stated as follows: given a network, or a graph, predict what edges will form between nodes in the future. Alternatively, in domains where data collection is costly and the resulting networks are noisy and incomplete, link prediction can be used to identify unobserved edges. In such cases, the problem is also known as the missing link problem.

The objective of this work is to better identify undiscovered (missing and suspicious) links between pairs of nodes in a protein-protein interaction (PPI) network. Link prediction uses the existing protein interaction topology to predict missing links. Discovery of links in biological networks such as gene networks, protein-protein interaction networks, metabolic networks etc. are very costly and time-consuming if done via laboratory experiments and hence the known connections within these networks remains largely incomplete (Martinez et al. 1999; Sprinzak et al. 2003). Instead of identifying links between all possible pairs of nodes, predictions that focus on already known interactions and are accurate enough can sharply reduce the experimental costs. Discovering protein protein interactions is a pivotal task for understanding the underlying biological processes behind tasks such as protein function prediction, drug delivery control and disease diagnosis.

Researchers have formulated link prediction as a binary classification problem, where class labels indicate the presence or absence of a link (referred to as positive links or negative links respectively) between pairs of nodes in the network. In this approch, features based on network topology such as common neighbors, Jaccard coefficient, etc. of the two nodes under consideration are fed to the classifier which predicts the presence or absence of a link. This paper also formulates the link prediction problem as a binary classification problem based on topological features, with a view to improve classification performance. Recently it was found that local community-based features were most effective for link prediction in biological networks both in monopartite (Cannistraci et al. 2013b) and bipartite networks (Daminelli et al. 2015). Therefore, we have included these in our feature list for the PPI network datasets under consideration.

An unresolved issue with formulating the link prediction problem as a classification problem is the label noise present in the training data. Typically, a set of positive and negative links are randomly chosen from the existing graph and are used for training a classifier, which is then used to predict links on the remaining network. However, the absence of a link in the network does not necessarily mean it is a negative link; it may be the case that the link exists but is undiscovered (as commonly occurs with PPI networks). Therefore, to include this pair of nodes in the training data as a negative link may introduce label noise and bias the resulting classifier. In this paper, we claim that by using anomaly detection on the negative links of the training data, and by subsequently filtering out the detected anomalous negative links from training data, we can obtain better classifiers that yield superior link prediction performance. The suggested approach is evaluated on five different PPI networks, with four different classifiers. A comparison with classification with and without anomaly detection is provided and results demonstrate that utilizing anomaly detection for filtering suspicious negative links yields superior classifier performance on test data.

## Related work

General purpose neighborhood based methods have been proposed for link prediction in different kinds of networks: collaboration, social, citation, roadmaps, etc. (Liben Nowell and Kleinberg 2007; Zhou et al. 2009). Various bio-inspired methods were created to either assess reliability of interactions in PPI networks such as Interaction Generality (IG1) (Saito et al. 2002), IG2 (Saito et al. 2003) and IRAP (Chen et al. 2005) or predict protein function such as the Czekanowski-Dice Dissimilarity (CDD) (Brun et al. 2003) and FSW (Chen et al. 2006). Later, these techniques were applied to protein interaction prediction (Cannistraci et al. 2013b; Chua et al. 2006). Both approaches rely on the number of neighbors that two non-directly connected nodes have and assign a likelihood score to this pair of nodes.

The simplest techniques are Jaccard’s coefficient (Jaccard 1912), Common Neighbors and Preferential Attachment (Newman 2001). Jaccard’s coefficient assigns higher likelihood scores to the node pairs for which the set of common interactors as a proportion of all available neighbors is higher and Common Neighbors does the same for pairs of nodes that simply share more interactors. Preferential Attachment, on the other hand, gives high scores when both nodes have a large number of neighbors: if one of the nodes has a low number of interactors, the score is reduced. In contrast, Adamic and Adar (2003) and Resource Allocation (Ou et al. 2007) are two similar indices that give more importance to Common Neighbors with low degree.

Various other methods have been proposed to assess the reliability of high-throughput protein interaction data. In 2009, Kuchaiev et al. (2009) proposed a method for geometric denoising of PPI networks. Cannistraci et al. in (2010) proposed topology-based link prediction method using minimum curvilinear embedding. In 2013, Cannistraci et al. (2013a) proposed a new valid variation of minimum curvilinear embedding, named non-centred minimum curvilinear embedding. Alanis-Lobato et al. in (2013) utilized several measures for the proximity of genes based on the common neighborhood structure of a GI network. However these methods do not explicitly utilize a classification based approach to the problem of identifying missing interactions.

Hasan et al. (2006) formulated the link prediction problem into binary classification problem. The method extracted a set of topological features of the network as input for supervised learning for link prediction. A binary classification approach integrated information from multiple measures to get a better prediction. In 2011 Fire M et al. (2011) utilized topological features for supervised learning, and ranked the importance of each feature. They proposed a set of simple, computationally efficient topological features that could be analyzed to identify missing links. In 2013 Cannistraci et al. (2013b) proposed a new paradigm to support link formation called the Local Community Paradigm (LCP), which emphasizes the role of the local network community structure in link formation. They proposed local community-based Cannistraci features for link-prediction in PPI networks. Yu et al. (2006) in 2006 predicted missing links in PPI networks by completing defective cliques. Some methods have been reviewed in Lü and Zhou (2011) and some have been successfully applied for link detection in PPI networks.

Several anomaly detection techniques have been proposed for detecting outlier nodes, edges or substructures in graph data. The techniques may broadly be classified as: i) Feature-based approaches which utilize structural graph-centric features for outlier detection in the constructed feature space. Essentially, these methods transform the graph anomaly detection problem to the well-understood outlier detection problem (Akoglu et al. 2010; Henderson et al. 2011). ii) Proximity-based approaches that exploit the graph structure to measure closeness (or proximity) of objects in the graph. These methods capture the simple autocorrelation between these objects, where similar objects are likely to belong to the same class (Jeh and Widom 2002; Brin and Page 1998). iii) Community-based approaches that utilize clustering methods for graph anomaly detection and rely on finding densely connected groups of ’close-by’ nodes in the graph to discover anomalies that have connections across communities (Chakrabarti 2004; Sun et al. 2005; Tong and Lin 2011). iv) Relational learning based approaches consist of network-based collective classification algorithms, the main idea of which is to exploit the relationships between the objects to assign them into classes, where the number of classes is often two: anomalous and normal (Getoor et al. 2001; Jensen et al. 2004). Further details on these approaches can be found in a thorough survey (Akoglu et al. 2015). In this paper we use feature-based anomaly detection techniques to discover suspicious negative links, thereby reducing the impact of label noise introduced by assigning undiscovered positive links to the class of negative links in the training data.

## Materials and methods

### Network Datasets

We used four protein-protein interaction (PPI) network datasets: Caenorhabditis elegans, Mus musculus, Arabidopsis thaliana and Rattus norvegicus. These are publicly available and were collected from the Protein Interaction Network Anaysis (PINA) platform. The platform integrates data from six curated databases and builds a complete, non-redundant dataset for the model organisms ^1^. Since, only interactions reported across multiple datasets were considered after careful curation, in this paper we assume that the reported interactions are relatively noise free. A brief summarization of the nework characteristics is provided in Table 1:

**Table 1:** Network datasets

Network	Type	No. of nodes	No. of edges
Arabidopsis thaliana	Undirected	7550	19962
Caenorhabditis elegans	Undirected	5758	14829
Mus musculus	Undirected	6236	13865
Rattus norvegicus	Undirected	2448	3804

### Methods

Our objective was to minimize the classification bias arising due to currently undiscovered edges (positive links) being incorrectly labeled as negative links. To address this bias, we use anomaly detection for removing suspicious negative links (which may be undiscovered positive links) from the training set before classifier training. Finally, we train a link classifier on the filtered dataset after removal of these detected suspicious negative links. Since we focused on predicting links based only on network topology, we extracted a set of features for node-pairs (edges) from the corresponding PPI network with the goal of developing a network topological feature-based classifier. We then performed supervised learning, using different machine learning classifiers. The network topology based features utilized for classification are described here.

#### Topology-based Measures

We briefly describe the set of topology-based measures or features that were used during our experiment. A graph theoretic approach is used to model the protein-protein interaction as a network. In this method, a PPI network is represented by an undirected graph *G*=(*V*,*E*), with a set of nodes or vertices *V* and a set of links or edges *E*, where vertices represent proteins and edges represent interactions between proteins respectively. In this paper, *G* will always be an un-weighted, undirected graph. Graphs can be characterized by many different topology-based measures, each one reflecting some particular traits of the studied structure. The topology-based measures were chosen based on their successful application in prior work on link prediction (Cannistraci et al. 2013b; Fire et al. 2011; Zhou et al. 2009).

**Node-based measures:** Let *N*(*v*) denote a neighborhood (or open neighborhood) of a node *v* in a graph *G*. *N*(*v*) is the set of all the nodes adjacent to *v*. The closed neighborhood of a node *v*, denoted by *N*[ *v*] is simply the set {*v*}∪*N*(*v*). The Formal definitions of neighborhoods that were used in this study to extract topological measures are:

1 $$ \begin{aligned} N(v) &= \{ u \mid (u,v) \in E \; or \; (v,u) \in E\}\\ N[\!v] &= N(v) \cup \{v\} \end{aligned}  $$

Based on the above definition, neighborhood-subgraph of *v* which induced by the neighborhoods of *v* are defined as:

2 $$ \begin{aligned} nbhd-subgraph(v)&=\{(x,y) \in E \mid x,y \in N(v)\}\\ nbhd-subgraph[\!v]&=\{(x,y) \in E \mid x,y \in N[\!v]\} \end{aligned}  $$

Note that the Induced subgraph of the open and closed neighborhoods of a node are very different with respect to their topological properties.Following measures for a node are created using the above neighborhood definitions:

- **Node degree :** The degree of a node in a network is the number of links the node has to other nodes. For an undirected network, degree of a node is defined as:
  Let *v*∈*V* and
  3 $$ deg(v)= |N(v)|  $$
- **Node subgraphs:** This measure denotes the number of links within the open and closed nbhd-subgraphs for each node *v*, which is defined as:
  4 $$ \begin{aligned} subgraph-edge-no(v) &= |nbhd-subgraph(v)|\\ subgraph-edge-no[\!v] &= |nbhd-subgraph[\!v]| \end{aligned}  $$
  Density of subgraph is defined as:
  5 $$ \begin{aligned} density-nbhd-subgraph(v)&= \frac {deg(v)} {nbhd-subgraph(v)}\\ density-nbhd-subgraph[\!v]&= \frac {deg(v)} {nbhd-subgraph[\!v]} \end{aligned}  $$

Note that the formal density of a graph is defined differently, however, the aim of this feature and all other features used in the paper is to be as straight forward and simple as possible. Therefore, we used a somewhat different density that is more related to a vertex *v*.

**Edge-based measures:** Let *u*,*v*∈*V* where *u*,*v*∉*E*. Using the neighborhoods of *u* and *v* we extract various measures. These measures help to determine the likelihood that a link between *u* and *v* exists.

- **Common-Neighbors (CN):** The common neighbors (CN) of *u* and *v* refers to the number of common neighbors of *u* and *v*. Two vertices *u* and *v* are more likely to connect if they have bigger number of common neighbors. It is defined as Newman (2001):
  6 $$ CN(u,v)=|N(u) \cap N(v)|  $$
- **Total-Neighbors (TN):** The total neighbors (TN) of *u* and *v* measure the number of distinct neighbors of *u* and *v*. which refers to the total number of neighbors *u* and *v* have together. The formal definition of TN is:
  7 $$ TN(u,v)=|N(u) \cup N(v)|  $$
- **Jaccard’s Coefficient (JC):** Jaccard’s coefficient (JC) normalizes the size of common neighbors by total neighbors. This gives higher weight to those pairs of nodes which share a higher proportion of common neighbors relative to the total number of neighbors they have. The formal definition of JC is (Jaccard 1912):
  8 $$ JC(u,v)=\frac{|N(u) \cap N(v)|}{|N(u) \cup N(v)|}  $$
- **Adamic-Adar Coefficient (AA):** This metric refines the simple counting of common neighbors by assigning higher likelihood scores to neighbors that are not shared with many others. It is defined as (Adamic and Adar 2003):
  9 $$ AA(u,v)=\sum_{z \in N(u) \cap N(v)} \frac{1}{log(|N(z)|)}  $$
- **Resource allocation Coefficient (RA):** The RA coefficient and AA coefficient have very similar forms the only difference being that the RA coefficient punishes the high degree common neighbors more heavily than the AA coefficient. It is defined as (Ou et al. 2007):
  10 $$ RA(u,v)=\sum_{z \in N(u) \cap N(v)} \frac{1}{|N(z)|}  $$
- **Preferential Attachment (PA):** This measure assigns higher likelihood scores to those pairs of nodes for which one or both nodes have a high degree. The formal definition of PA is (Newman 2001):
  11 $$ PA(u,v)=|N(u)|.|N(v)|  $$
- **LCP-based measures and Cannistraci variants:** The local community paradigm suggests that two nodes are more likely to link together if their common-first-neighbors are members of a strongly inner-linked cohort or local-community. The Cannistraci (LCP-based) variants of classical neighborhood methods (CN, PA, AA, RA, JC) are defined as (Cannistraci et al. 2013b):
  12 $$\begin{array}{@{}rcl@{}} CAR(u,v)= CN(u,v).LCL(u,v) =CN(u,v).\sum_{z \in N(u) \cap N(v)}\frac{|\gamma(z)|}{2} \end{array} $$
  13 $$\begin{array}{@{}rcl@{}} CPA(u,v)= e_{u}.e_{v} + e_{u}.CAR(u,v) + e_{v}.CAR(u,v) + CAR(u,v)^{2} \end{array} $$
  14 $$\begin{array}{@{}rcl@{}} CAA(u,v)=\sum_{z \in N(u) \cap N(v)}\frac{|\gamma(z)|}{{log}_{2}(|N(z)|)} \end{array} $$
  15 $$\begin{array}{@{}rcl@{}} CRA(u,v)=\sum_{z \in N(u) \cap N(v)}\frac{|\gamma(z)|}{|N(z)|}\!\!\qquad\qquad\qquad\qquad\qquad\quad \end{array} $$
  16 $$\begin{array}{@{}rcl@{}} CJC(u,v)=\frac{CAR(u,v)}{|N(u) \cup N(v)|}\!\!\qquad\qquad\qquad\qquad\qquad\quad \end{array} $$
  Where *γ*(*z*) refers to the sub-set of nodes in the neighborhood of *z* that are also common neighbors of of *u* and *v*, thus |*γ*(*z*)| is the local community degree of *z*; *e*_*u*_ refers to the external degree of *u*, and is computed considering the nodes in the neighborhood of *u* that are not common neighbors of *u* and *v*.
- **Friends Measure (FM):** Friend Measure (FM) of *u* and *v* measures the total number of links between the neighborhoods of *u* and *v*. Here we assume that two nodes have higher chance to get connected if their neighborhoods have more links with each other. The formal definition of FM is (Fire et al. 2011):
  17 $$ FM(u,v)=\sum_{x \in N(u)} \sum_{y \in N(v)} \delta(x,y)  $$
  Where
  18 $$ \delta(x,y)= \left\{\begin{array}{cl} 1 & if\ x=y \;or\; (x,y) \in E \;or\; (y,x) \in E\\ 0 & otherwise \end{array}\right.  $$

**Edge Subgraph-based measures:** The following subgraphs are defined by using the neighborhoods definitions (Fire et al. 2011):Let *u*,*v*∈*V*

19 $$ \begin{aligned} nbhd-subgraph(u,v)=\{(x,y) \in E \mid (x,y \in N(u) \cup N(v))\}\\ nbhd-subgraph[\!u,v]=\{(x,y) \in E \mid (x,y \in N[\!u] \cup N[\!v])\} \end{aligned}  $$

The above subgraph equations contain information about the number of links between the neighborhood of *u* and *v* including the inner connections or links between each node neighborhood. The following subgraph equation represents the inner-connection subgraph:

20 $$ \begin{aligned} inner-subgraph(u,v)=\{(x,y) \in E \mid (x \in N(u) \; and \; y \in N(v)) \; or \; \\ (y \in N(u) \; and \; x \in N(v))\} \end{aligned}  $$

- **Edge Subgraphs Edges Number:** This measure counts the number of links in the above subgraphs:
  21 $$ \begin{aligned} |nbhd-subgraph(u,v)|\\ |nbhd-subgraph[\!u,v]|\\ |inner-subgraph(u,v)| \end{aligned}  $$

In this study, we extracted a total of 25 features for each PPI network.

#### Anomaly detection

We attempt to apply multiple anomaly detection techniques such as Parzen Windows, Principal Component Analysis (PCA), Nearest Neighbor (a distance-based method) and a one-class Gaussian process for removing anomalous negative links from the training data. The details of these methods can be found in (Clifton 2007; 2009; Pimentel et al. 2014). We utilize the link prediction feature set for training the anomaly detector, described in an earlier subsection. Note that all the methods presented below require only normal data for training, however abnormal data is used for validating the models. In that sense the methods below may be considered unsupervised. as these methods do not require anomalous data for training. After experimentation, we found that the Gaussian Process based anomaly detection gave the most reliable results. Hence, we chose the Gaussian Process model as our anomaly detector for our classification experiments. Next, we present a brief introduction to all of the methods considered.

**Parzen window method:** The Parzen window kernel density estimator method (Parzen 1962) is the model adopted here to estimate the probability density function (pdf), p(x), for the training (normal) data. With this method (Bishop 2006), *p*(*x*) is estimated using the following steps:

1. Locate a hyperspherical Gaussian window, or kernel, with width *σ*, on each of the D-dimensional feature vectors in the training dataset, *x*_*i*_, where i = 1, …, N.
2. Evaluate the sum of the Gaussian distributions using the squared Euclidean distances between the test feature vector *x* and the training vectors *x*_*i*_, normalized by a factor that ensures *p*(*x*) integrates to 1.

This gives the following formula for the estimate of *p*(*x*):

22 $$ p(x)=\frac{1}{N(2\pi)^(D/2)(\sigma)^{D}} \times exp\left(-\frac{|| x - x_{i}||^{2}} {2.\sigma^{2}}\right)  $$

By placing a Gaussian kernel over each feature vector *x*_*i*_ in our training dataset, we construct a probability density estimate of *p*(*x*) that will have a higher value of *p* where the concentration of training data is greatest. Points in the test set with values of *p*(*x*) are classified as anomalies.

**PCA method:** PCA is an orthogonal transformation for transforming the raw data into a space such that the new basis vectors (principal components) are linear combinations of the original basis vectors, are linearly uncorrelated and correspond to the directions of maximal variance of the data, where the first principal component is in the direction of the highest variance, the second in the direction of the highest remaining variance and so on. Anomaly detection is performed with PCA under the assumption that normal data would be best explained by looking at the first few principal components whereas abnormal data would be captured by the remaining principal components (Bishop 2006; Chiang et al. 2001; Marsland 2003). Thus points in the data that have high coefficients for the last few principal components would correspond to anomalous data.

**Nearest neighbor method:** These approaches rely on the intuition that normal points will have normal neighbours in their vicinity and abnormal points would conversely have fewer normal points in their neighborhood (Hautamäki et al. 2004). Assuming that normal data is partitioned into clusters, the Novelty score z(x) of a data point x for some cluster width *σ*_*k*_ k is given by:

23 $$ z(x)= \frac{1}{\sigma_{k}} \lVert x, \mu_{k} \rVert_{2}  $$

where,

$$\sigma_{k} = \sqrt{\frac{1}{N_{k}} \sum_{j \in k} (x_{j} - \mu_{k})^{2}}$$

and *μ*_*k*_ is the centre of cluster k, and *σ*_*k*_ is defined to be the standard deviation of intra-cluster distances (Clifton 2009). Now, points with high Novelty scores for all clusters are regarded as anomalous.

**Gaussian process:** Given a training set $D =\{(x_{i},y_{i})\}_{i=1}^{n} =(X,y)$ where *x*_*i*_∈*X*⊂*R* ^*d*^ denotes feature vector and y denotes a scalar output or target. We are interested in identifying the target *y*_∗_ for a new sample *x*_∗_. The objective of regression is to find the association between inputs *x* and target *y*. To identify the association between the input and target, we modelled the mapping in terms of *y*=*f*(*x*)+*ε*, where *f* is an unknown function, and *ε* denotes a noise term. To do this, one approach is to assume that *f* is a parametric function *f*(*x*;*θ*) where the parameters *θ* are tuned based on the training data. But, the major pitfall of this kind of approach is that, if in case, a wrong form of the function is chosen, it can lead to poor predictions. Another approach, based on Gaussian process takes care of this problem by assigning a priori probability to all possible functions, which are more likely to be sampled. The process is based on the assumption that these functions are drawn from a specified probability distribution. This method requires a training set and may be considered supervised.

The core of GP regression lies in the selection of a prior probability distribution over latent function which are sampled from a Gaussian process i.e., $f \sim \mathcal {GP} (m(x),\kappa (x,x'))$. Where *m*(*x*) and *κ*(*x*,*x* ^′^) are mean and covariance function respectively. Without any prior knowledge about the underlying data, the most common choice is to choose a GP with mean zero. Gaussian Process can be described as a generalization of multivariate Gaussian distribution, where the dimensions can extend to infinity. The latent function *f* is said to follow a Gaussian process, if and only if every finite subset of function values is multivariate Gaussian distributed. Therefore, the function values *f* obey the model below:

24 $$ f \mid X \sim \mathcal{N} (m(X),\kappa(X,X))  $$

Furthermore, we assume the noise *ε* to be Gaussian distributed with mean zero and standard deviation *σ*_*n*_ i.e., $\epsilon \sim \mathcal {N} (0,\sigma _{n}^{2})$. As a result, now output value *y*_∗_ for test sample *x*_∗_ can be deduced in a Bayesian manner by marginalizing over latent function *f*. Given training data *D*, the predictive distribution of *y*_∗_ is normally distributed i.e.,

25 $$ y_{*} \mid D,x_{*} \sim \mathcal{N} (\mu_{*},\sigma_{*}^{2})  $$

Where moments *μ*_∗_ and $\sigma _{*}^{2}$ can be given in closed form expressions. More details about GP framework, can be found in (Williams and Rasmussen 2006).

In 2010, Kemmler et al. (2010) have shown how GP regression can be employed for one-class classification problems. They proposed using both the predictive mean *μ*_∗_ (GP-Mean) and negative variance $-\sigma _{*}^{2}$ (GP-Var) as one-class scores applied to training data with labels y = 1:

26 $$ \mu_{*} = k_{*}^{T}(K + \sigma_{n}^{2}.I)^{-1}.1  $$

27 $$ - \sigma_{*}^{2} = -(k_{**} - k_{*}^{T}(K + \sigma_{n}^{2}.I)^{-1}k_{*} + \sigma_{n}^{2})  $$

Where *K*=*κ*(*X*,*X*) denotes the kernel matrix of the training set, *k*_∗_=*κ*(*X*,*x*_∗_) represents the vector of kernel values between training set and test input and *k*_∗∗_=*κ*(*x*_∗_,*x*_∗_) is the kernel values of the test input. The correlation of function values using the similarity of input samples are calculated by the radial basis function (rbf): $\kappa (x,x')=exp\left (-\frac {|| x - x^{'}||^{2}} {2.\sigma ^{2}}\right)$.

#### Experimental setup

Since the number of known links are few, we oversample the positive links in the datasets to generate sufficient positive links from each network when required. The set of negative links is much larger and it has been shown that the subset sampling method used to generate the negative training links impacts the performance of the resulting classifier (Yu et al. 2010). Two predominant sampling methods have been proposed for the negative set sampling in PPI networks, namely balanced random sampling and simple random sampling. In simple random sampling care is taken to ensure the proteins in the positive set must also appear in the negative set. In balanced random sampling the proteins must occur with the same frequency in both sets. It has further been shown that protein pairs with higher number of common neighbours are more likely to interact (Alanis-Lobato 2015), therefore by choosing non interacting pairs within 2 hops of each other we are in effect constructing a negative set that is harder to classify. To ensure no bias is introduced due to the sampling method we experimented with both balanced random and simple random sampling for choosing our negative set.

**Fig. 1:**
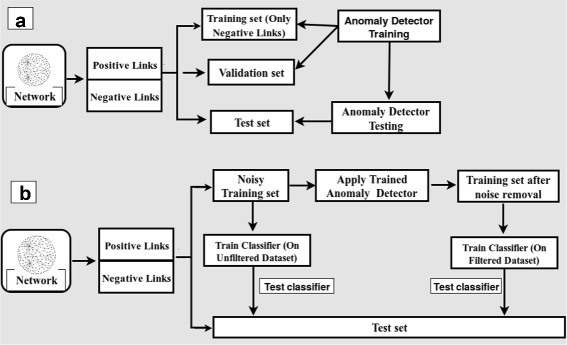
Experimental setup overview. **a** Training and Testing of Anomaly Detection Methods. **b** Classifiers prediction on the unfiltered and filtered dataset

Figure 1 shows the workflow of the proposed method. The methodology is configurable into two phase. In the first phase, we construct a dataset to train the anomaly detector to filter out the anomalous negative links from training data of each PPI network. In the second phase, we construct another dataset (disjoint from the dataset in Phase-I) to train a classifier to classify a pair of nodes as a positive link or a negative link for each PPI network. To this end, we construct first and second phase as follows:

**Phase-I:** In this phase, First, we construct the dataset to train the anomaly detector. Then, we train and evaluate performance of different anomaly detection methods on each PPI network.

1. We extract positive links from the network, and divided them into a validation and test set in the ratio 50:50. Note that positive links are not used for training the anomaly detector but only for validation.
2. We extract negative links from the network, such that the vertices are within two hops of each other. These are divided into training, validation and test set in the ratio 60:20:20.
3. Topological features are extracted for the above training, validation and test sets.
4. We train and evaluate different anomaly detection methods and select the best performing anomaly detection method from these methods. We use this trained anomaly detector model in phase-II.

**Phase-II:** In this phase, We construct another dataset to train the classifiers. Then, we train and evaluate the performance of different classifiers on each PPI network.

1. We extract positive links from the network, and divided them into a training set, validation set and a test set in the ratio 60:20:20.
2. In order to introduce synthetic noise we mislabel a fraction of the positive links and assign them labels corresponding to negative links.
3. We extract negative links from the network using simple random sampling, such that the vertices are within two hops of each other.
4. The mislabelled negative links generated in step 2 are merged with the negative links in the training set from step 3 to allow for creation of a noisy dataset with synthetic ground truth. This is divided into a training, validation and test set in the ratio 60:20:20 such that the positive and negative datasets are balanced.
5. Topological features are extracted for the above training and test sets. We call this the unfiltered training dataset.
6. Next we generate a filtered version of the dataset using anomaly detection to filter out the noisy negative links we had generated in step 4.
7. We evaluate different machine learning classifiers on both the filtered and unfiltered training datasets.
8. Prediction accuracy is compared across classifiers trained on the filtered vs. the unfiltered dataset.

## Results

### Performance evaluation of different anomaly detector

We trained different anomaly detection methods on the training set of each PPI network and measured the performance on corresponding test set, where the training and test sets are constructed as described in Phase-I of the previous subsection. We trained the anomaly detection methods on negative links (normal class) only and utilized the positive links (abnormal class) for validation and test purpose. Each method yields an anomaly score on the validation set and a threshold is chosen for detection based on minimizing the false positive and false negative rate on the validation set. We report accuracy metrics on the test set using the chosen optimal threshold in Table 2. We notice that One Class Gaussian Process (gpoc) anomaly detection technique has a better score than the other anomaly detectors. Since this was consistent across the datasets, hence in this paper we used One Class Gaussian Process technique for anomaly detection.

**Table 2:** Anomaly detection techniques comparison under different metrics

Network	Anomaly Method	Accuracy	F-measure	Sensitivity	Specificity	FP rate	FN rate
Arabidopsis	gpoc	96.69	96.77	99.19	94.19	5.81	0.81
thaliana	parzen	85.75	85.12	81.50	90.00	10.00	18.50
	pca	69.25	76.48	1	38.50	61.50	0
	nn	77.50	81.63	1	55.00	45.00	0
Caenorhabditis	gpoc	90.98	91.59	98.23	83.73	16.27	1.77
elegans	parzen	69.63	76.61	99.50	39.75	60.25	0.50
	pca	56.13	69.50	1	12.25	87.75	0
	nn	56.37	69.63	1	12.75	87.25	0
Mus musculus	gpoc	94.90	95.13	99.56	90.24	9.76	0.44
	parzen	90.62	91.39	99.50	81.75	18.25	0.50
	pca	70.37	77.15	1	40.75	59.25	0
	nn	76.50	80.97	1	53.00	47.00	0
Rattus	gpoc	98.10	98.13	99.68	96.52	3.48	0.32
norvegicus	parzen	96.13	96.25	99.50	92.75	7.25	0.50
	pca	77.25	81.47	1	54.50	45.50	0
	nn	91.37	92.06	1	82.75	17.25	0

Now we focus our attention on the dataset described earlier for Phase-II. We apply the anomaly detector trained above only on negative links of noisy Phase-II training set. We validate the performance of the GP anomaly detector (one class gaussian process) using True Positive Rate (TPR) and True Negative Rate (TNR). Results are provided in Table 3. The TNR is slightly lower than TPR because of uncertain labels of negative links i.e. some of the negative links may be positive links and may be detected as outliers.

**Table 3:** Anomaly detector performance measure

Network	TPR	TNR
Arabidopsis thaliana	99.62	93.23
Caenorhabditis elegans	99.22	82.33
Mus musculus	99.70	91.50
Rattus norvegicus	99.65	95.58

### Gene Ontology (GO) validation of anomaly detection

We also elucidated the biological significance of anomaly detection using the Gene Ontology (GO) scores of protein pairs in different PPI networks. Since proteins which are involved in the same biological function or share the same biological pathway are more likely to interact with each other compared to proteins which belong to other pathways, hence this statistics is a better measure to test the quality of our prediction. We calculated the GO score corresponding to each of the gene ontology classes i.e. biological process, cellular components and molecular functions of protein pairs using the Protein Interaction Network Analysis Platform (PINA) ^2^. As we saw in Table 3 TNR is lower than the TPR this is because our anomaly detector extracts some non interacting protein pairs as anomalies. These may be undiscovered interactions and to validate this hypothesis we look at the GO scores of these anomalous protein pairs. In Mus musculus, the anomaly detection extracts 1360 proteins pairs as anomalies out of which 612 protein pairs have a GO score greater than 0.5, and out of these 268 protein pairs were found to interact with different public databases. In Rattus norvegicus, 707 protein pairs were extracted out of which 543 protein pairs had GO scores greater than 0.5. In Caenorhabditis elegans, 2826 protein pairs were extracted as anomalies out of which 1254 protein pairs had GO scores greater than 0.5. Thus, a high proportion of the protein pairs filtered by the anomaly detection technique outlined in this paper appear to have significant GO scores and may potentially have undiscovered interactions. We further validated the discovered anomalies against the Negatome Database which contains experimentally supported non-interacting protein pairs. On matching the resuts not a single anomalous negative link discovered by the anomaly detector was found to lie in the Negatome database, further validating the fact that the interactions discovered by the anomaly detector.

### Performance evaluation of different classifiers

After we removed the anomalies and generated the two training sets before and after filtering out the suspicious negative links, we trained four standard classifiers on both training sets. We evaluated the different machine learning classifiers (SVM, C5.0, KNN and Naive Bayes) on each PPI network. We used three standard metrics Accuracy, F-measure and Area Under the ROC curve (AUC) to measure the performance of each classifier. The F-measure indicates the trade off between precision and recall score of a classifier for a particular threshold setting whereas the AUC is independent of the threshold. It is an evaluation of the classifier as threshold varies over all possible values. In evaluation terminology, we denote the set of true positives as TP, the set of true negatives as TN, the set of false positives as FP, the set of false negative as FN. Various evaluation metrics is defined as:

28 $$ Recall \quad or \quad True \enspace Positive \enspace Rate \quad or \quad Sensitivity = \frac{TP}{TP+FN}  $$

29 $$ True \enspace Negative \enspace Rate \quad or \quad Specificity = \frac{TN}{TN+FP}  $$

30 $$ Precision = \frac{TP}{TP+FP}  $$

31 $$ Accuracy = \frac{TP+TN}{TP+TN+FP+FN}  $$

32 $$ F-measure=\frac{2*Precisions*Recall}{ Precisions + Recall}  $$

33 $$ False \enspace Positive \enspace Rate = \frac{FP}{FP+TN}  $$

34 $$ False \enspace Negative \enspace Rate = \frac{FN}{FN+TP}  $$

As mentioned earlier we experiment with both simple random and balanced random sampling for constructing our negative set, please refer to Tables 4 and 5 for a comparitive analysis. Results indicate that the accuracies of the models do not change significantly using either approach, so for all further experiments we chose simple random sampling as our sampling method.

**Table 4:** Classification comparison Under different metrics using simple random sampling

Network	Classifier	Without anomaly detection			With anomaly detection		
		Accuracy	F-measure	AUC	Accuracy	F-measure	AUC
Arabidopsis	SVM	93.07	92.80	93.07	96.85	96.94	96.85
thaliana	C5.0*	99.35	99.35	99.35	99.34	99.34	99.34
	KNN	94.24	93.97	94.24	98.42	98.44	98.42
	NB	62.29	40.54	62.29	84.22	82.24	84.22
Caenorhabditis	SVM	87.67	86.02	87.67	94.02	94.34	94.02
elegans	C5.0	97.81	97.78	97.81	98.30	98.32	98.30
	KNN	92.99	92.73	92.99	96.07	96.20	96.07
	NB	59.18	36.39	59.18	67.23	57.46	67.23
Mus musculus	SVM	93.30	93.03	93.31	96.83	96.93	96.84
	C5.0	98.06	98.04	98.06	99.32	99.33	98.32
	KNN	94.12	93.84	94.12	98.47	98.49	98.47
	NB	60.38	35.23	60.38	79.32	75.55	79.32
Rattus	SVM	91.65	90.79	91.65	98.79	98.81	98.79
norvegicus	C5.0	91.34	90.54	91.34	99.46	99.45	99.45
	KNN	88.15	86.57	88.15	99.35	99.35	99.35
	NB	66.29	49.32	66.29	84.25	82.00	84.25

where * shows the *p*-value >.05

**Table 5:** Classification comparison Under different metrics using balanced random sampling

Network	Classifier	Without anomaly detection			With anomaly detection		
		Accuracy	F-measure	AUC	Accuracy	F-measure	AUC
Arabidopsis	SVM	92.99	92.70	92.99	97.01	97.09	97.01
thaliana	C5.0*	99.41	99.76	99.41	99.41	99.41	99.41
	KNN	94.81	94.60	94.81	98.43	98.45	98.43
	NB	62.55	41.18	62.55	86.77	85.53	86.77
Caenorhabditis	SVM	87.02	85.18	87.02	93.60	93.97	93.60
elegans	C5.0*	97.77	97.74	97.77	97.95	97.97	97.95
	KNN	92.63	92.33	92.63	96.20	96.32	96.20
	NB	60.32	41.53	60.32	66.90	56.98	66.90
Mus musculus	SVM	93.71	93.49	93.71	96.64	96.75	96.64
	C5.0	98.63	98.62	98.63	99.37	99.37	99.37
	KNN	93.14	92.74	93.14	98.38	98.41	98.38
	NB	59.67	33.26	59.67	78.60	74.78	78.60
Rattus	SVM	94.61	94.35	94.61	98.56	98.57	98.56
norvegicus	C5.0	90.75	89.84	90.75	99.40	99.40	99.40
	KNN	86.20	84.04	86.20	99.37	99.37	99.37
	NB	67.42	51.85	67.42	82.73	80.13	82.73

where * shows the *p*-value >.05

**Fig. 2:**
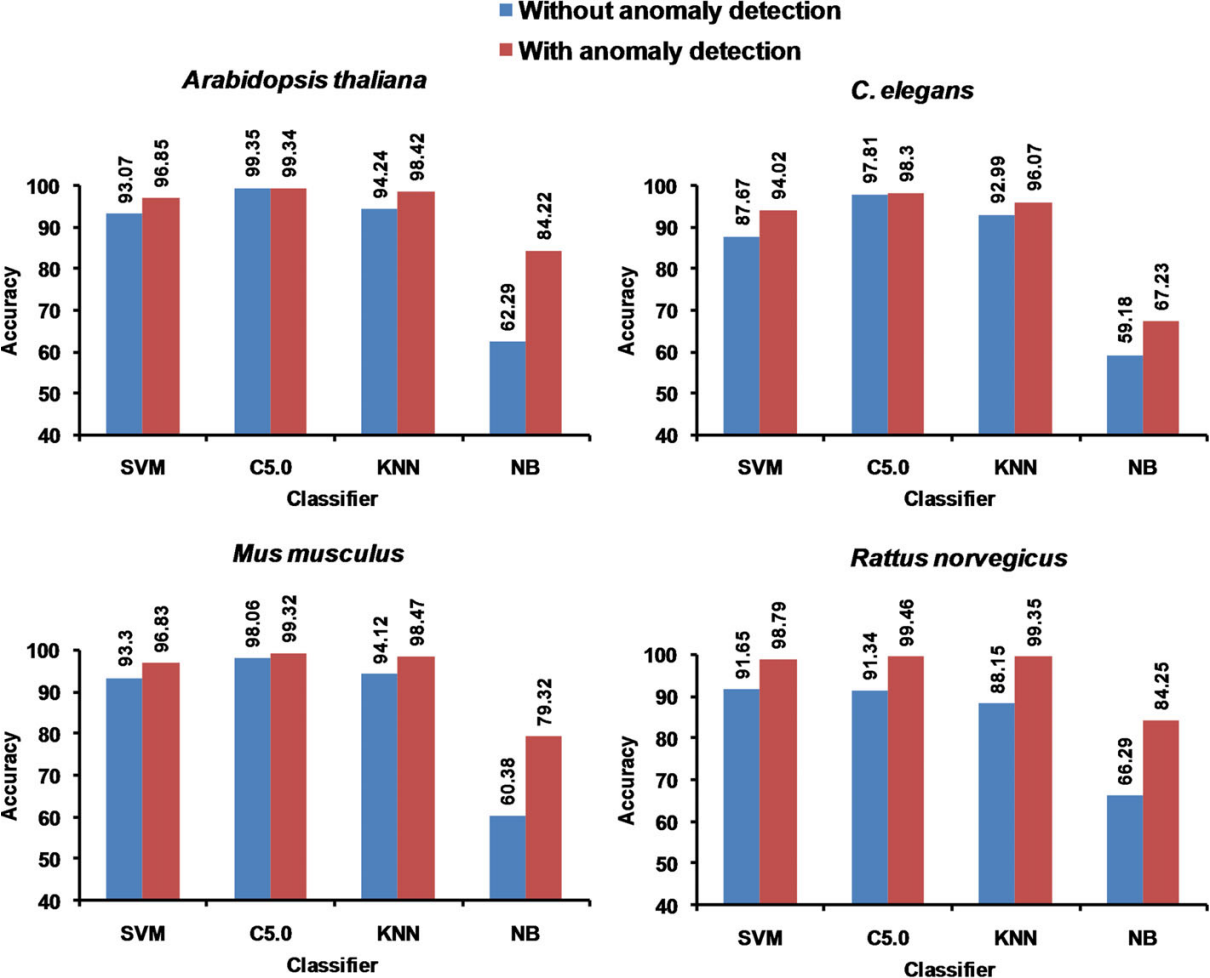
Accuracy Comparison of different classifiers with and without anomaly detection technique using simple random sampling

We repeat our experiments ten times by randomly selecting the training and test set to remove any statistical bias. Then we used the t-test for validating the statistical significance of differences between the Accuracy scores obtained with and without anomaly detection. In all cases barring that of the C5.0 classifier for the Arabidopsis thaliana dataset, we get a *p*-value < 0.0001 which shows that this difference may be considered to be extremely statistically significant. It can be seen that naive Bayes classifier is a weak classifier without anomaly detection technique, but improves most significantly after using anomaly detection technique. The reason for this is that the Naive Bayes classifier has more room for improvement after filtering the data via anomaly detection due to prior poor performance. The remaining classifiers SVM, C5.0, and KNN exhibit good classification performance before anomaly detection, but these too show significant performance improvement when anomaly detection is used for filtering the training set. The sole exception is the C5.0 classifier which yields 99.36 *%* accuracy on the Arabidopsis thaliana dataset without anomaly detection and therefore has very little margin for improvement after anomaly detection (this is marked with a * in Table 4). A comparison of the accuracies of the different classifiers on each PPI network is shown in Fig. 2. The results shown in Table 4 illustrate the classification performance measures in terms of Accuracy, F-measure, and AUC. In Table 4, we can see that all three performance measures (Accuracy, F-measure, AUC) improve with anomaly detection. Thus, it appears that classification performance improves after filtering via anomaly detection.

### Feature importance

In order to understand the contribution from each feature for link prediction in the PPI network, we comparatively analyzed the predictive power of the features. To measure the relative importance of different features, we analysed the information gain with respect to each feature. Information gain is based on the decrease in entropy after a dataset is split on an attribute. An attribute with highest information gain is selected for the split. We obtained the information gain of an attribute as follows: Information gain=(Entropy of distribution before the split) - (entropy of distribution after the split)

Where, entropy of a discrete probability distribution *p* on the countable set {*x*_1_,*x*_2_,*x*_3_,...}, with *p*_*i*_=*p*(*x*_*i*_), is defined as:

35 $$ h(p)= - \sum_{i \geq 1} p_{i}*log(p_{i})  $$

By comparing the entropy before and after the split, we obtain a measure of information gain (Han and Kamber 2006). Now, we ranked all the features based on its information gain. Table 6 presents the Information gain on the training sets of all the PPI networks. It can be seen that Common-Neighbors, Adamic Adar Coefficient, Resource allocation Coefficient, Cannistraci-based Preferential Attachment, Friends Measure, number of links in inner-subgraph and number of links in neighborhoods-subgraph are higgly influential for almost all of the PPI networks. In PPI networks, we know that proteins that form complexes display common functions. So, if proteins A, B, and C share the same function and protein A interacts with B and C, it is very probable that B and C also interact. Thus it is expected that Common-Neighbors would be an influential feature for link prediction in PPI networks (Alanis-Lobato 2015). We also know that proteins which are grouped together into cliques and quasi-cliques in PPI networks share identical functions and hence have greater probability of link formation in a densely connected group of proteins. The number of links in inner-subgraph and in neighborhood-subgraph are thus also highly influential features for link prediction in PPI networks. It is also noteworthy that more nuanced neighbor counting features like Adamic-Adar and Resource Allocation are more predictive than features that rely only on the number of common neighbors.

**Table 6:** InfoGain values of different features for all PPI networks

	Networks						
	Arabidopsis	Caenorhabditis	Mus	Rattus		Average	Standard
	thaliana	elegans	musculus	norvegicus		deviation	
deg(u)	0.32	0.10	0.24	0.260		0.23	0.09
deg(v)	0.24	0.093	0.17	0.17		0.17	0.06
subgraph-edge-no(u)	0.23	0.07	0.19	0.20		0.17	0.07
subgraph-edge-no(v)	0.18	0.07	0.13	0.14		0.13	0.05
subgraph-edge-no[u]	0.32	0.11	0.23	0.25		0.23	0.09
subgraph-edge-no[v]	0.24	0.09	0.16	0.17		0.17	0.06
density-nbhd-subgraph(u)	0.27	0.11	0.23	0.23		0.21	0.07
density-nbhd-subgraph(v)	0.23	0.10	0.17	0.17		0.17	0.05
density-nbhd-subgraph[u]	0.28	0.11	0.23	0.23		0.21	0.07
density-nbhd-subgraph[v]	0.23	0.10	0.17	0.17		0.17	0.05
**CN(u,v)**	**0.56**	**0.60**	**0.54**	**0.58**		**0.57**	**0.03**
TN(u,v)	**0.52**	0.20	0.41	0.49		0.40	0.15
JC(u,v)	0.08	0.03	0.037	0.12		0.06	0.04
**AA(u,v)**	**0.82**	**0.73**	**0.79**	**0.80**		**0.79**	**0.04**
**RA(u,v)**	**0.82**	**0.71**	**0.79**	**0.80**		**0.78**	**0.05**
PA(u,v)	**0.54**	0.23	0.42	**0.51**		0.42	0.14
CAR(u,v)	0.08	0.06	0.11	0.09		0.08	0.02
**CPA(u,v)**	**0.67**	0.40	**0.59**	**0.76**		**0.60**	**0.15**
CAA(u,v)	0.10	0.07	0.11	0.09		0.09	0.02
CRA(u,v)	0.11	0.08	0.12	0.10		0.10	0.02
CJC(u,v)	0.08	0.06	0.10	0.09		0.08	0.02
**FM(u,v)**	**0.82**	**0.58**	**0.69**	**0.66**		**0.69**	**0.10**
|**nbhd-subgraph(u,v)** |	**0.71**	0.40	**0.55**	**0.61**		**0.57**	**0.13**
|nbhd-subgraph[u,v] |	**0.50**	0.19	0.36	0.44		0.37	0.14
|**inner-subgraph(u,v)** |	**0.86**	**0.61**	**0.72**	**0.71**		**0.72**	**0.10**

All features with average deviation (or average mean) > 0.5 for all networks are in bold. All values > 0.5 are in bold

### Performance evaluation across datasets

To evaluate the generalization of our method across different datasets we conducted experiments where a model trained on one dataset is tested on all the other datasets. Tables 7, 8, 9 and 10 tabulate the results for models trained on Arabidopsis thaliana, Caenorhabditis elegans, Mus musculus and Rattus norvegicus datasets respectively. The results demonstrate that for the most part the models derived from topological features on one dataset using anomaly detection show a gain over models learned without anomaly detection. The one exception is the translation of performance to C elegans which suggests that this network may be topologically somewhat different from the rest. Interestingly, the models trained on C elegans with anomaly detection do exhibit a strong gain in performance on other datasets. Overall though the method does seem to translate well across datasets.

**Table 7:** Results for Classifier trained on Arabidopsis thaliana and tested on remaining datasets

Classifier	Networks	Without anomaly detection			With anomaly detection		
		Accuracy	F-measure	AUC	Accuracy	F-measure	AUC
SVM	Rattus norvegicus	96.71	96.64	96.71	98.98	98.99	98.98
	Mus-Musculus	93.78	93.68	93.78	95.79	95.96	95.79
	C.elegans	85.03	86.03	85.03	81.63	84.48	81.63
C5.0	Rattus norvegicus	99.46	99.46	99.46	99.45	99.45	99.45
	Mus-Musculus	99.34	99.34	99.34	99.22	99.22	99.22
	C.elegans	96.92	97.00	96.92	95.72	95.89	95.72
KNN	Rattus norvegicus	96.66	96.56	96.67	99.58	99.58	99.58
	Mus-Musculus	95.45	95.31	95.45	98.18	98.20	98.18
	C.elegans	91.38	91.45	91.37	90.52	91.34	90.52
NB	Rattus norvegicus	64.42	44.95	64.42	77.33	71.09	77.33
	Mus-Musculus	60.78	36.47	60.78	76.14	70.10	76.15
	C.elegans	57.96	35.03	57.96	70.69	68.43	70.69

**Table 8:** Results for Classifier trained on Caenorhabditis elegans and tested on remaining datasets

Classifier	Networks	Without anomaly detection			With anomaly detection		
		Accuracy	F-measure	AUC	Accuracy	F-measure	AUC
SVM	Rattus norvegicus	81.24	76.99	81.24	99.05	99.05	99.05
	Mus-Musculus	82.57	78.98	82.57	98.23	98.25	98.23
	A.thaliana	81.28	77.04	81.28	97.93	97.95	97.93
C5.0	Rattus norvegicus	95.56	95.37	95.56	97.87	97.83	97.87
	Mus-Musculus	95.07	94.82	95.07	98.48	98.46	98.48
	A.thaliana	94.77	94.49	94.77	98.68	98.66	98.68
KNN	Rattus norvegicus	93.94	93.56	93.94	99.08	99.08	99.08
	Mus-Musculus	93.45	93.02	93.45	98.53	98.52	98.53
	A.thaliana	93.29	92.87	93.29	98.29	98.29	98.29
NB	Rattus norvegicus	65.38	47.24	65.38	74.37	65.78	74.37
	Mus-Musculus	62.56	40.95	62.56	71.66	61.22	71.66
	A.thaliana	62.62	40.82	62.62	72.99	64.02	72.99

**Table 9:** Results for Classifier trained on Mus musculus and tested on remaining datasets

Classifier	Networks	Without anomaly detection			With anomaly detection		
		Accuracy	F-measure	AUC	Accuracy	F-measure	AUC
SVM	Rattus norvegicus	96.93	96.86	96.93	99.02	99.02	99.02
	C.elegans	83.96	84.87	83.96	81.52	84.40	81.52
	A.thaliana	92.83	92.67	92.83	95.66	95.84	95.66
C5.0	Rattus norvegicus	97.93	97.89	97.93	99.68	99.68	99.68
	C.elegans	94.64	94.69	94.64	95.80	95.96	95.80
	A.thaliana	97.13	97.06	97.13	99.37	99.37	99.37
KNN	Rattus norvegicus	94.64	94.36	94.64	99.72	99.43	99.72
	C.elegans	91.50	91.63	91.50	91.68	92.31	91.68
	A.thaliana	93.43	93.12	93.43	98.26	98.29	98.26
NB	Rattus norvegicus	65.63	47.83	65.63	79.26	74.37	79.26
	C.elegans	57.06	33.21	57.06	69.10	67.95	69.10
	A.thaliana	62.28	40.61	62.28	81.72	79.44	81.72

**Table 10:** Results for Classifier trained on Rattus norvegicus and tested on remaining datasets

Classifier	Networks	Without anomaly detection			With anomaly detection		
		Accuracy	F-measure	AUC	Accuracy	F-measure	AUC
SVM	Mus-Musculus	90.89	90.68	90.89	92.81	93.29	92.81
	C.elegans	77.71	80.01	77.71	74.33	79.57	74.33
	A.thaliana	90.91	90.76	90.91	92.57	93.07	92.57
C5.0	Mus-Musculus	84.24	81.46	84.24	98.61	98.62	98.61
	C.elegans	82.43	79.73	82.43	94.31	94.59	94.31
	A.thaliana	82.03	78.25	82.03	98.75	98.75	98.75
KNN	Mus-Musculus	84.38	81.85	84.38	96.63	96.72	96.63
	C.elegans	81.45	79.86	81.45	85.88	87.57	85.88
	A.thaliana	83.11	80.19	83.11	96.57	96.66	96.57
NB	Mus-Musculus	65.93	50.25	65.93	85.73	84.82	85.73
	C.elegans	60.12	48.44	60.12	71.98	74.68	71.98
	A.thaliana	68.10	56.24	68.10	87.29	87.10	87.29

## Discussion

This paper presents a technique for filtering graphical link training data by using anomaly detection for the purpose of link prediction in PPI networks. The performance of the resulting predictor compares favourably with the classifier trained on unfiltered data. The central idea is to have a filtering step before the classification step where suspicious links are removed from the training data. One issue that needs emphasis here is that the choice of anomaly detection technique plays a critical role in the success of the resulting classifier. If the anomaly detector is inaccurate, then the classifier may not yield optimum performance. One way to ascertain the efficacy of the anomaly detection is by deliberately mislabeling the positive links and checking if the anomaly detection algorithm can detect them, which is how we have selected our Gaussian Process algorithm. Additionally, this technique shows most improvement in performance when the link prediction accuracy is not particularly high before filtering, as this allows for greater room for classification improvement. Additionally, this technique needs to be extended to link prediction in networks with directed edges (metabolic networks), weighted edges (neural networks). While the given technique is useful for detecting missing links more efficiently, it may have to adapt to work for evolving networks where the links are constantly changing. These ideas are the focus of our future work.

## Endnotes

^1^ Downloaded from: http://cbg.garvan.unsw.edu.au/pina/interactome.stat.doon February 10, 2015.

^2^ http://cbg.garvan.unsw.edu.au/pina/interactome.goSimForm.do
